# Molecular Mechanistic Pathways Targeted by Natural Products in the Prevention and Treatment of Alcoholic Liver Disease

**DOI:** 10.3390/life16030375

**Published:** 2026-02-26

**Authors:** Kaixuan Zhou, Jincai Li, Menghu Wang, Mingjun Yu, Jing Wang

**Affiliations:** 1 College of Traditional Chinese Medicine, Bozhou University, Bozhou 236800, China; 2 College of pharmacy, Changchun University of Chinese Medicine, Changchun 130117, China

**Keywords:** alcoholic liver disease, natural products, oxidative stress, inflammation, lipid deposition, mechanism

## Abstract

Alcoholic liver disease (ALD) is a condition caused by alcohol abuse. Although its incidence is rapidly increasing, effective treatments for this disease remain limited. The pathogenesis of ALD involves multiple aspects, such as direct damage from alcohol, oxidative stress, inflammation, lipid metabolism disorders, and dysbiosis of the gut microbiota. Under the combined action of multiple damaging factors, liver inflammation worsens and leads to liver fibrosis. In this review, we focused on in vitro and in vivo experiments to investigate the mechanistic pathways by which natural products exert effects against the progression of ALD. The accumulated and collected data indicate that some natural products may regulate oxidative stress, inflammation, autophagy, lipid metabolism, and the intestinal barrier, thereby protecting the liver. This review presents an updated overview of the potential benefits of these natural products for the prevention and treatment of ALD, identifying potential new therapeutic lead compounds and providing a reference for innovative drug development and clinical treatment for ALD.

## 1. Introduction

Alcoholic liver disease (ALD) is liver damage caused by long-term excessive alcohol consumption. It initially manifests as significant hepatocellular steatosis, which can progress to steatohepatitis, liver fibrosis, and cirrhosis. Severe short-term alcohol abuse can also lead to acute severe alcoholic hepatitis, chronic acute liver failure, and even death [1,2]. Compared to non-drinkers, drinkers have a significantly higher risk of developing cardiovascular diseases and cancers. Furthermore, heavy drinkers face significantly increased risks at all stages, from the onset of disease to death [3]. Many factors affect ALD, including alcohol consumption, the duration of alcohol consumption, the variety of alcoholic beverages, gender, race, obesity, hepatitis virus infection, genetic factors, and nutritional status [4]. Complete abstinence from alcohol is the most fundamental treatment for alcoholic liver disease, and patients should be provided with nutritional support to reduce the severity of alcoholic liver disease and treat alcohol-induced cirrhosis and its complications [5].

In the treatment of ALD, metadoxine can accelerate the clearance of alcohol from serum, improve the symptoms of alcoholism, and reduce alcohol dependence and behavioral abnormalities [6]. Glycyrrhizin preparations, silybin, and glutathione demonstrate varying degrees of antioxidant, anti-inflammatory, and hepatoprotective effects. Their clinical application can reduce liver injury-related indicators such as ALT, AST, GGT, and TG [7]. In recent years, some studies have shown that certain natural products have anti-oxidative and anti-inflammatory effects, which are beneficial in the treatment of liver disease [8,9]. In this review, we aim to provide the most recent and comprehensive insights into in vitro and in vivo investigations of natural products for the treatment of ALD and highlight potential mechanisms and molecular targets, especially for signaling pathways involved in metabolic regulation and anti-oxidation, anti-inflammatory, and anti-fibrosis effects, identifying potential new therapeutic lead compounds and providing a reference for innovative drug development and clinical treatment for ALD.

## 2. Pathogenesis of ALD

**Figure 1:**
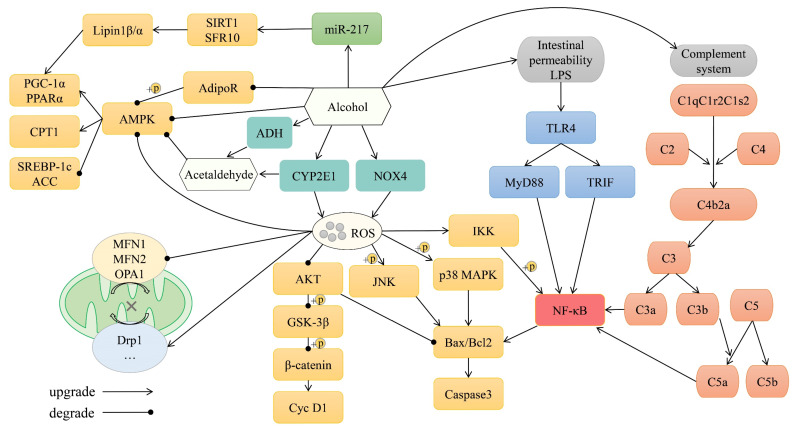
Pathophysiological mechanisms of ALD.

The pathogenesis of ALD is complex and may include (1) direct injury by alcohol, (2) injury by the alcohol metabolite acetaldehyde, (3) oxidative stress, (4) mitochondrial damage, (5) lipid metabolism disorder, (6) inflammation, (7) apoptosis, (8) dysbiosis of the gut microbiota, and (9) complement disorders [10,11]. The pathogenesis of ALD is illustrated in Figure 1. There are two pathways of ethanol metabolism in the body. In one pathway, alcohol dehydrogenase (ADH) metabolizes ethanol to produce acetaldehyde, which is then metabolized to non-toxic acetic acid by acetaldehyde dehydrogenase (ALDH). In the other pathway, ethanol is metabolized by cytochrome P450 2E1 (CYP2E1), which converts it to acetaldehyde; notably, this pathway produces significant amounts of reactive oxygen species (ROS) [12]. Long-term drinking will increase the expression and activity of CYP2E1, and CYP2E1 will produce excessive ROS even in the absence of a substrate [13,14]. NADPH oxidase 4 (NOX4) is a NADPH oxidase expressed in hepatocytes, and excessive alcohol activates NOX4 receptors, resulting in increased intracellular ROS production [15]. This increase in ROS production promotes the phosphorylation of c-Jun N-terminal kinase (JNK) and p38 mitogen-activated protein kinase (MAPK), inhibits the phosphorylation of protein kinase B (Akt), and then increases the Bax/Bcl-2 ratio and Caspase-3 expression, promoting hepatocyte apoptosis [16,17]. Appropriate activation of the Akt/Glycogen synthase kinase-3β (GSK-3β) pathway is crucial for energy metabolism and cell survival in the liver. The inhibition of Akt phosphorylation by ROS increases downstream GSK-3β phosphorylation, leading to phosphorylation and degradation of β-catenin, resulting in downregulation of cyclin D1 and cell cycle arrest [18,19]. Excessive ROS promote the expression and phosphorylation of IκB kinase (IKK), which promotes the expression and phosphorylation of nuclear factor kappa-B (NF-κB), thereby enhancing inflammation [20,21]. Mitochondria play an important role in energy metabolism and lipid oxidation in the liver. Excess ROS produced by long-term drinking can induce mitochondrial permeability transition and DNA damage, resulting in increased expression of mitochondrial fission-related protein dynamin-1-like protein (Drp1) and decreased expression of the mitochondrial fusion-related proteins mitofusin-1 (Mfn1), Mfn2, and optic atrophy protein 1 (OPA1), leading to cell apoptosis [22,23]. Alcohol, acetaldehyde, and ROS can directly inhibit the phosphorylation of AMP-activated protein kinase (AMPK), thus increasing the expression of sterol regulatory element-binding protein 1c (SREBP-1c) and acetyl-CoA carboxylase (ACC) and decreasing the expression of carnitine O-palmitoyltransferase 1 (CPT1), peroxisome proliferator-activated receptor gamma coactivator 1α (PGC-1α), and peroxisome proliferator-activated receptor α (PPARα), ultimately reducing fatty acid oxidation and increasing fatty acid synthesis [24,25,26,27]. Adiponectin is a crucial hormone that is synthesized and secreted by adipose tissue, and it plays a significant role in the regulation of lipid metabolism within the liver. Adiponectin interacts with AdipoR in the liver, thereby affecting lipid metabolism through AMPK and other signaling pathways. Alcohol can inhibit adiponectin secretion by adipocytes and the expression of AdipoR in the liver, leading to lipid metabolism dysregulation [28,29]. Lipin-1 is a major participant in the development of fatty liver disease. Lipin-1β is mainly involved in lipid synthesis in the cytoplasm, while lipin-1α acts as a transcriptional co-activator in the nucleus to promote PGC-1α and PPARα expression. Alcohol can inhibit the expression of miR-217, reduce the expression of sirtuin 1 (SIRT1) and the splicing factor arginine/serine-rich 10 (SFRS10), and thus increase the lipin-1β/α ratio [30,31].

Gut microbial homeostasis is crucial for health. Excessive alcohol consumption can compromise this balance by increasing intestinal permeability and disrupting the microecology, which adversely alters the microbial composition by reducing beneficial bacteria and promoting harmful ones. For instance, it can increase the relative abundance of potential pathogens like *Enterococcus*, *Parabacteroides*, and *Alistipes* and decrease that of *Akkermansia* [9,32]. Lipopolysaccharide (LPS) produced by the gut microbiota enters the body’s circulation through the damaged intestinal barrier and binds to toll-like receptor 4 (TLR4), activating the downstream myeloid differentiation primary response 88 (MyD88)/NF-κB and TIR domain-containing adapter molecule 1 (TRIF)/NF-κB signaling pathways, thereby aggravating liver inflammation [33,34,35]. Complement is an important part of the innate immune system and plays a crucial role in immune response and the maintenance of various metabolic reactions. Most complement components are produced in the liver. Alcohol can activate the C1 complex (C1qC1r2C1s2), which leads to the cleavage of C2 and C4, generating the C3 convertase (C4b2a). The C3 convertase then cleaves C3 into C3a and C3b. Subsequently, C3b contributes to the formation of the C5 convertase, which finally cleaves C5 into C5a and C5b. When C3a and C5a bind to their respective receptors, the TLR4/NF-κB pathway can be activated, leading to the release of inflammatory cytokines [36,37]. Figure 1 summarizes the pathogenesis of ALD.

## 3. Compounds for the Treatment of ALD

### 3.1. Flavonoids

Quercetin, a representative flavonoid, is present in numerous fruits and vegetables. It demonstrates good pharmacological activity for the treatment of liver diseases [38]. Many studies have shown that quercetin can significantly improve liver injury caused by alcohol. Chen et al. investigated the role of quercetin through its interaction with glycinamide ribonucleotide transformylase in the purine metabolism pathway. The results showed that quercetin regulated this pathway, reduced the expression of aging markers (p53, p21, and p16), and alleviated ethanol-induced lipid deposition and cellular senescence in hepatocytes [39]. Zeng et al. used a Lieber–DeCarli liquid diet to establish an ALD model in C57BL/6J mice, and quercetin was used for treatment. In the alcohol-fed group, ethanol constituted 36% of the total caloric intake (approximately 5% *v*/*v* ethanol in the final diet). The pair-fed control group received an isocaloric diet in which ethanol-derived calories were isocalorically replaced with maltose dextrin. This model was employed to simulate chronic and metabolic alcohol consumption in humans, effectively inducing hepatic steatosis and liver injury while minimizing the stress and gastrointestinal disturbances associated with forced intragastric administration. The results showed that quercetin significantly alleviated alcohol-induced liver injury and lipid deposition. Quercetin promotes liver lipophagy by activating AMPK, increasing LC3II protein expression, and decreasing perilipin-2 (PLIN2) protein levels [40]. Yu et al. found that quercetin could increase the expression of AMPK, extracellular signal-regulated kinase 2 (ERK2), Parkin, and voltage-dependent anion-selective channel protein 1 (VDAC1) and promote forkhead box protein O3 (FOXO3a) nuclear translocation, thereby enhancing mitophagy and alleviating alcohol-induced liver mitochondrial damage [41]. In addition, quercetin alleviated lipid deposition and the production of reactive oxygen species in the mitochondria of alcohol-injured L02 cells, increased the mRNA levels of mitochondrial fusion genes (OPA1 and MFN1), and inhibited the mRNA levels of mitochondrial fission genes (MFF, Drp1, and Fis1), thus restoring the dynamic mitochondrial balance. Quercetin also promoted PGC-1α nuclear translocation and inhibited NLRP3 inflammasome activation, alleviating pyroptosis [42]. Quercetin has also been observed to inhibit the NF-κB/NLRP3 inflammasome pathway by increasing the expression of nuclear factor erythroid 2-related factor 2 (Nrf2)/heme oxygenase 1 (HO-1), alleviating liver inflammation and oxidative stress caused by alcohol [43]. Liu et al. used the Lieber–DeCarli liquid diet to establish an ALD model in C57BL/6N mice and generated frataxin knockdown or overexpression in HepG2 cells, which were injured by alcohol. Their results showed that quercetin could alleviate the liver histopathological changes and mitochondrial dysfunction caused by alcohol and regulate the expression of PTEN-induced putative kinase protein 1 (PINK1), Parkin, BCL2/adenovirus E1B 19 kDa protein-interacting protein 3 (BNIP3), LC3II, p62, TOM20, and VDAC1, thereby improving mitophagy. In addition, quercetin regulated the intracellular trivalent iron level and increased the expression of frataxin, thereby alleviating mitochondrial dysfunction [44]. Some researchers have also observed mitochondrial damage and iron deposition in alcohol-injured hepatocytes. Alcohol causes endoplasmic reticulum (ER) stress by activating the PERK signaling pathway, resulting in mitochondrion-associated endoplasmic reticulum membrane damage. At the same time, alcohol reduces Gpx4 and xCT and increases long-chain-fatty-acid–CoA ligase 4 (ACSL4) in hepatocytes, indicating the occurrence of ferroptosis. The administration of quercetin reversed these processes, protected mitochondrial function, and reduced iron deposition and endoplasmic reticulum stress in the liver [45]. In addition to reducing iron deposition, quercetin counteracted ethanol-induced changes in key autophagy markers: it decreased p62 and increased the levels of nuclear receptor coactivator 4 (NCOA4) and LC3II. Furthermore, it prevented the colocalization of NCOA4 with ferritin heavy chain and the nuclear translocation of forkhead box protein O1 (FOXO1), ultimately modulating ferritinophagy [46]. Zhao et al. treated alcohol-soaked zebrafish larvae with quercetin, finding that quercetin significantly alleviated oxidative stress and liver lipid deposition in zebrafish in a dose-dependent manner through a mechanism related to the regulation of the P2X7r-mediated PI3K/Keap1/Nrf2 signaling pathway [47]. Although quercetin is well-tolerated and safe in humans and available as a nutritional supplement in tablet or powder form, with a typical daily dose of 500 to 1000 mg, its clinical application is limited by several pharmacokinetic drawbacks: quercetin’s poor aqueous solubility, limited intestinal absorption, rapid metabolism in the body, and short half-life all contribute to its low bioavailability. To address this, incorporating quercetin into a nanoformulation has been proposed as a promising strategy to enhance its solubility, promote intestinal absorption, and improve targeting [48,49].

Cyanidin-3-O-β-glucoside (C3G) is the most widely distributed anthocyanin in edible fruits. It has many biological activities, such as anti-inflammatory, antioxidant, lipid-lowering, and neuroprotective effects and can improve glucose metabolism [50,51]. Some studies have shown that C3G can improve ALD. C3G can reduce lipid deposition in the livers of ALD mice, enhance the level of liver antioxidant enzymes, and maintain the integrity of the intestinal barrier. C3G improved disturbances in gut microbiota caused by alcohol, restored the ratio of *Firmicutes* to *Bacteroidetes* at the phylum level, and improved the abundance of *Muribaculaceae*, *Bacteroides*, *Blautia*, *Collinsella*, *Escherichia-Shigella*, *Ruminococcus*, *Enterococcus*, *Prevotella*, *Romboutsia*, *Streptococcus*, *Bilophila*, and *Methylobacterium-Methylorubrum* at the genus level [52]. He et al. found that C3G demonstrated multifaceted protective effects in ALD mice. It not only alleviated hepatic lipid deposition and inflammation while reducing SAA1 expression but also ameliorated oxidative stress by downregulating CYP2E1 and decreasing 4-HNE and MDA levels. Additionally, C3G enhanced autophagy and mitophagy by modulating the AMPK pathway to regulate the protein levels of PINK1, Parkin, LC3II, and TOM20 [53]. Zhou et al. found that C3G inhibited the acetylation of NF-κB by upregulating the expression of SIRT1, which inhibited the activation of the NLRP3 inflammasome, thereby improving lipid deposition and inflammation in the livers of ALD mice [54]. Although C3G has potential benefits for ALD, its low stability and bioavailability limit the efficacy and distribution of C3G in the human body. Conjugates made with lipids, polysaccharides, proteins, and nanocapsules can facilitate targeted delivery and may be used to improve C3G’s bioavailability [55]. The toxicity of C3G also requires further research. An early study showed that protocatechuic acid, the main metabolite of C3G, was toxic to the liver and kidneys of mice at a high dose (500 mg/kg, i.p.), resulting in glutathione depletion and abnormal liver and kidney function indicators [56]. Table 1 summarizes the available data on flavonoids’ activities and the mechanisms of their action.

### 3.2. Alkaloids

Capsaicin is an alkaloid that produces the spicy flavor in chili peppers. Studies have found that it has anti-inflammatory and lipid-lowering effects, with potential therapeutic effects in hepatitis and atherosclerosis [57,58]. Early studies found that a diet containing capsaicin could reduce acute alcohol-induced lipid accumulation in rat livers, and capsaicin reduced the serum ethanol concentration in a mouse model of chronic alcohol consumption [59]. Koneru et al. found that capsaicin increased the activity of liver antioxidant enzymes, inhibited the expression of NF-κB, and restored the activity of mitochondrial respiratory enzymes and the balance of matrix metalloproteinase (MMP)/tissue inhibitor of metalloproteinase (TIMP) in the liver, thus alleviating liver injury [60]. Capsaicin is a highly selective agonist of transient receptor potential cation channel subfamily V member 1 (TRPV1). A study found that capsaicin can activate ESCRT-III-dependent membrane repair by activating TRPV1 on the cell membrane and Ca^2+^ influx, thereby alleviating alcohol-induced pyroptosis of hepatocytes [61]. Capsaicin, the principal active component in chili peppers, is generally considered safe. Its acute oral LD_50_ has been determined to be 161.2 mg/kg in rats and 118.8 mg/kg in mice [62]. To date, most clinical research on capsaicin has concentrated on its topical application for managing chronic neuropathic pain [63]. Consequently, further investigation is required to evaluate its efficacy and potential for treating ALD.

Berberine is a well-known natural isoquinoline alkaloid derived from traditional Chinese herbal plants, including *Coptis chinensis* and *Berberis poiretii*, with anti-inflammatory, antioxidant, and metabolism-supporting effects [64]. Berberine has a protective effect against liver injury induced by alcohol, mitigating oxidative stress and lipid deposition in the liver. This mechanism involves the inhibition of CYP2E1 and the reactivation of the PPARα/PGC-1α/hepatocyte nuclear factor 4-alpha (HNF4A)/microsomal triglyceride transfer protein large subunit (MTTP) pathway [65]. Ke et al. found that berberine attenuated levels of inflammatory factors in the blood and hepatic lipid deposition in ALD rats. Berberine significantly decreased the expression of thyroid hormone-inducible hepatic protein (THRSP), fatty acid synthase (FASN), ACC, and ATP-citrate synthase (ACLY) in the liver and upregulated the expression of PPARα, CPT1α, and peroxisomal acyl-coenzyme A oxidase 1 (ACOX1), thereby reducing lipid synthesis and increasing lipid oxidation [66]. Zhu et al. found that berberine binds well to AMPK. It activates the AMPK/SIRT1 pathway, increasing the expression of SREBP-1c, SREBP-2, FASN, and HMG-CoA reductase (HMGCR), which can improve lipid metabolism disorders in ALD [67]. Berberine also contributes to the improvement of ALD by regulating the gut microbiota. At the phylum level, berberine significantly enhanced *Verrucomicrobia*, of which the sole identified member is *Akkermansia muciniphila*, indicating a potential clinical application in the prevention and treatment of diabetes and obesity. At the genus level, populations of *Terrisporobacter* and *Helicobacter* increased following berberine treatment, whereas *Pseudoflavonifractor*, *Mucisirillum*, *Alistipes*, *Ruminiclostridium*, and *Lachnoclostridium* decreased [68]. Berberine has achieved a high degree of clinical translation, and its use is supported by numerous high-quality clinical studies. It has been approved for the treatment of diarrhea in some countries (e.g., China), and substantial evidence-based medical evidence has accumulated for its application in managing metabolic diseases such as diabetes and hyperlipidemia [69,70]. Berberine exhibits a favorable safety profile with infrequent adverse effects, which may occasionally include nausea, vomiting, rash, and drug fever. Interestingly, its extremely low oral bioavailability (typically <1%) results in high intestinal concentrations. This enables berberine to exert its core pharmacological action: modulating the gut microbiota [71]. Given these properties, berberine is a promising candidate compound for treating ALD. Future research should prioritize the validation of its clinical efficacy, in-depth elucidation of its mechanisms of action, and optimization of berberine formulations to support innovative applications in liver disease therapy. Table 2 summarizes the available data on alkaloid activities and the mechanisms of their action.

### 3.3. Terpenoids

Glycyrrhizic acid is a pentacyclic triterpenoid saponin compound derived from *Glycyrrhiza* species with antioxidant, anti-inflammatory, and hepatoprotective effects. It is commonly used in the clinical treatment of drug-induced liver injury and viral hepatitis [72,73]. Liu et al. found that glycyrrhizic acid can reduce lipid deposition and pyroptotic bodies in the livers of ALD mice. In their in vitro study, glycyrrhizic acid alleviated the lipid deposition of the THP-1 and HepG2 co-culture system damage by FFA and ethanol. Glycyrrhizic acid regulates the SHP1/SYK signaling pathway in macrophages, inhibiting hepatic lipid peroxidation and pyroptosis [74]. Diammonium glycyrrhizinate, a pharmaceutical form of glycyrrhizic acid, demonstrated hepatoprotective effects. Its mechanism involves upregulating dead-box helicase 5 (DDX5), which reduces signal transducer and activator of transcription 1 (STAT1) phosphorylation, thereby inhibiting oxidative stress and inflammation. Additionally, diammonium glycyrrhizinate alleviates hepatic lipid deposition by downregulating key lipogenic genes, including FASN, stearoyl-CoA desaturase1(SCD1), and SREBP-1c [75]. Glycyrrhizic acid has the epimers 18α- and 18β-glycyrrhizic acid. Studies have shown that the optimal ratio of 18α- and 18β-glycyrrhizic acid for improving inflammatory cytokines and chemokines in ALD rat models is 4:6 [76]. Clinically, glycyrrhizic acid is primarily used to treat liver diseases. However, attention must be paid to its toxic effects. Glycyrrhetinic acid, the intestinal metabolite of glycyrrhizic acid, is a potent inhibitor of 11-β-hydroxysteroid dehydrogenase. This inhibition leads to abnormally elevated cortisol levels, which in turn can cause water and sodium retention, hypertension, and hypokalemia [77]. Long-term use may result in edema and cardiac complications.

Asperosaponin VI is a triterpenoid saponin compound derived from the *Dipsacus asper* Wall. Studies have reported anti-inflammatory, antioxidant, and anti-apoptotic effects of asperosaponin VI, which have beneficial effects on metabolic diseases such as diabetes, atherosclerosis, and non-alcoholic fatty liver disease [78]. Wei et al. found that asperosaponin VI can improve ALD. In vivo, asperosaponin VI alleviated oxidative stress, inflammation, lipid deposition, and fibrosis in the livers of ALD mice. In vitro, asperosaponin VI alleviated lipid deposition in alcohol-injured HepG2 cells. Asperosaponin VI’s ability to improve ALD is related to the regulation of the AMPK and pancreatic eIF2-alpha kinase (PERK)/E74-like factor 2 (ELF2) signaling pathways, which improve lipid metabolism and ER stress in hepatocytes [79]. Despite preliminary evidence indicating the therapeutic potential of asperosaponin VI for ALD, its clinical development is hindered by extremely low oral bioavailability owing to its poor gastrointestinal permeability and extensive degradation before absorption. To advance its development, structural modifications are needed to enhance bioavailability, coupled with further toxicological and clinical studies [80].

Hederagenin is a pentacyclic triterpenoid saponin that is somewhat enriched in *Hedera nepalensis* var. *sinensis*. Studies have shown that hederagenin has various pharmacological activities that can be applied to treat hyperlipidemia, including hepatoprotective and anti-inflammatory effects [81]. It also has a potential therapeutic effect on ALD: hederagenin has been shown to improve blood lipids and alleviate liver damage in rats with ALD, improve the expression of alcohol-metabolizing enzymes, and reduce the expression of inflammation-related (TNF-α, IL-6, and COX-2) and apoptosis-related (Bax/Bcl-2 p53) proteins in the liver. Its mechanism of action is related to the activation of the Akt and ERK signaling pathways [82]. Hederagenin is a natural compound precursor with a broad spectrum of pharmacological activity that has not yet entered clinical development. Its efficacy and clinical translation are primarily hindered by its low oral bioavailability and complex in vivo metabolic processes. Furthermore, attention must be paid to the potential hemolytic activity associated with saponin compounds [83]. Table 3 summarizes the available data on terpenoid activities and their mechanisms of action.

### 3.4. Phenylpropanoids

Nodakenin is a furanocoumarin glycoside extracted from *Angelicae gigas*. It has anti-inflammatory effects and can improve lipid metabolism [84], and studies have shown that it has a potential therapeutic effect in ALD. Nodakenin can reduce activation of the NLRP3 inflammasome by activating the Nur77/P2X7r signaling pathway, thus reducing the inflammation and pyroptosis of hepatocytes. In addition, nodakenin also improved the expression of PPARα, SREBP-1, and Lipin-1 in the liver, alleviating hepatic lipid deposition [85]. Research on nodakenin remains at the preclinical stage, with existing studies concentrating on its pharmacodynamics and pharmacokinetics. Data from systematic toxicological evaluations and human clinical trials are still lacking, and its potential application in treating ALD necessitates further experimental validation.

Glycycoumarin is a coumarin compound isolated from *Glycyrrhiza uralensis* with significant pharmacological activities, including immunomodulatory, anti-inflammatory, and liver-protective effects [86]. Studies have shown that glycycoumarin can regulate the expression of LC3II and p62 in ALD model animals or cells, thereby increasing hepatocyte autophagy. In addition, glycycoumarin activates the p38 MAPK/Nrf2/HO-1 signaling pathway, improving oxidative stress in hepatocytes and thus alleviating liver injury [87]. Glycycoumarin is a natural coumarin with confirmed hepatoprotective activity and low preclinical toxicity. Pharmacokinetic studies indicate that after administration, glycycoumarin is widely distributed in tissues but accumulates predominantly in the liver, suggesting potential liver-targeting properties. Its development is limited by its pharmacokinetic characteristics, including a low oral bioavailability of only 13.82% in rats and a short half-life. Furthermore, glycycoumarin acts as an inhibitor of the metabolic enzyme UGT1A9, indicating a potential risk for drug–drug interactions when co-administered with drugs that are primarily metabolized by this enzyme [86,88].

α-Asarone is a simple phenylpropanoid in *Acorus tatarinowii* Schott, which exerts beneficial nerve-protecting and anti-inflammatory effects [89]. Studies have found that α-asarone can alleviate liver injury caused by alcohol. It increases the levels of liver antioxidant enzymes, alleviates oxidative stress and inflammation, reduces apoptosis-related protein p53, and increases the expression of liver autophagy proteins by activating the AMPK signaling pathway [90]. As multiple studies have indicated that α-Asarone and its isomers may be carcinogenic, mutagenic, and genotoxic [91], future research should prioritize systematic and long-term toxicological evaluation in addition to exploring their pharmacological activities in order to clarify the risk–benefit ratio. Table 4 summarizes the available data on phenylpropanoids’ activities and the mechanisms of their action.

### 3.5. Steroids

Dioscin is a natural steroid saponin present in many members of Dioscoreaceae. Pharmacological studies have shown that dioscin has anti-inflammatory, anti-hyperlipidemic, and hepatoprotective effects [92]. Liu et al. found that dioscin alleviated the levels of inflammatory (NF-κB) and fibrotic (αSMA, Collagen I) proteins by regulating the TLR4/MyD88 and transforming growth factor beta (TGF-β)/Smad signaling pathways in the livers of ALD mice, thereby improving liver inflammation and fibrosis [93]. Dioscin can increase liver antioxidant enzymes and reduce oxidative stress in ALD mice. Ethanol exposure also induces endoplasmic reticulum stress, which increases endoplasmic reticulum chaperone BiP (GRP78), ATF6, and eIF2, promotes the activation of the MAPK pathway, and increases the expression of inflammatory (NF-κB) and apoptotic (p53, Caspase-3, Caspase-9, and PARP) proteins. Dioscin was observed to alleviate the endoplasmic reticulum stress induced by ethanol and improve liver inflammation and apoptosis. It also regulated the expression of lipid metabolism-related proteins such as PPARα, medium chain acyl-CoA dehydrogenase (MCAD), ACSL1, ACSL5, ACADS, and ALDH7A1, thus improving liver lipid deposition [94]. In clinical practice, some drugs in which dioscin is the main component are used to treat cardiovascular diseases. However, the absolute oral bioavailability of dioscin is only 0.2%. Some researchers have incorporated dioscin into novel nano drug delivery systems to improve its oral bioavailability. In addition to its benefits, dioscin may demonstrate hepatotoxicity; the level of ALT increased significantly after the administration of dioscin at 300 mg/kg for 90 days. This effect deserves special attention in clinical experimental research [95,96].

Daucosterol is a phytosterol glycoside commonly found in plants, which has anti-inflammatory and immunomodulatory effects [97]. Research has shown that daucosterol may exert therapeutic effects in ALD. Daucosterol activates the Nrf2 pathway to improve alcohol-induced oxidative stress in liver cells and can effectively reduce the upregulation of alcohol-induced lipid synthesis (FASN, SREBP-1c, and SCD1) and fibrotic (Collagen1, Collagen 3A1, and αSMA) proteins, thereby alleviating lipid accumulation and liver fibrosis. Daucosterol also improves alcohol-induced liver inflammation by regulating inflammasome activation through the p38 MAPK/NF-κB pathway [98]. There have been no toxicological, pharmacokinetic, or clinical trials of daucosterol as of yet. Computer simulation studies have predicted that it has good pharmacokinetic characteristics, but these findings need to be verified in rigorous experimental studies [99]. Table 5 summarizes the available data on steroid activities and the mechanisms of their action.

### 3.6. Other Compounds

Ligustilide is a phthalide derived from *Angelica sinensis* (Oliv.) Diels and *Ligusticum chuanxiong* Hort., with anti-aging, anti-tumor, analgesic, and other biological activities [100,101,102]. Yang et al. found that ligustilide has a potential therapeutic effect in ALD. In the livers of ALD mice, ligustilide significantly regulated the expression of PPARα and SREBP-1, effectively improving liver lipid deposition caused by alcohol. In addition, ligustilide reduced the expression of relaxin receptor 1 (RXFP1) and inhibited the activation of the NLRP3 inflammasome, alleviating pyroptosis. In in vitro studies, ligustilide has also alleviated lipid deposition in alcohol-injured AML12 cells by regulating PPARα and SREBP-1. In LPS/ATP-stimulated murine peritoneal macrophages (MPMs), ligustilide decreased the expression of RXFP1, inhibited the activation of NLRP3, and reduced the production of inflammatory factors [103]. A cellular safety assessment indicated that ligustilide exhibits low cytotoxicity, with IC_50_ values greater than 100 µM against both hepatocytes and neuronal cells, suggesting a minimal toxicity risk at conventional application levels [104]. However, its therapeutic application is significantly constrained by its unfavorable pharmacokinetics. Ligustilide has an extremely low absolute oral bioavailability of merely 2.6%, primarily due to extensive hepatic first-pass metabolism. Following intravenous administration, it shows a large volume of distribution but an exceptionally short elimination half-life (approximately 0.31 h) [105]. To overcome these limitations, future research should focus on the development of novel delivery systems and more comprehensive safety evaluations.

*Poria cocos* polysaccharide (PCP) is a complex component of *Poria cocos*, a fungus of *Polyporaceae*. PCP is mainly composed of myoinositol, sorbitol, galactose, glucose, mannose, and fucose, which have anti-inflammatory, antioxidant, hepatoprotective, neuroregulatory, and gut microbiota regulatory effects [106,107]. Huang et al. systematically researched the effects of PCP in a mouse model of ALD. The results indicated that PCP modulated the TLR4/ NF-κB signaling pathway, which alleviated liver inflammation and regulated the expression of genes involved in lipid metabolism, thereby reducing liver lipid deposition. PCP also enhanced intestinal barrier integrity and reshaped the gut microbiota composition. At the genus level, PCP treatment promotes the relative abundance of *Parabacteroides*, particularly *Parabacteroides distasonis*. Furthermore, PCP restored bile acid homeostasis, including levels of chenodeoxycholic acid and cholic acid, by regulating the FXR/FGF15/CYP7A1 signaling pathway [108]. Zhou et al. constructed in vivo and in vitro models of ALD and simultaneously set ferroptosis and Nrf2 inhibitor control. Their study showed that PCP increases the expression of the Nrf2 signaling pathway, increases the expression of ferritin heavy chain (FTH1) and Gpx4, and inhibits the NF-κB signaling pathway, thereby alleviating oxidative stress, inflammation, and ferroptosis caused by alcohol [109]. Jiang et al. found that PCP significantly alleviated alcohol-induced liver injury, lipid deposition, and oxidative stress. The mechanism of PCP’s therapeutic effect relates to regulating the TLR4/NF-κB signaling pathway to reduce inflammation and regulating the MAPK signaling pathway to reduce apoptosis. PCP also protects the intestinal barrier, reduces the entry of LPS produced by the gut microbiota into blood circulation, and reduces liver inflammation [110]. In 2015, an oral solution of PCP was approved by China’s National Medical Products Administration as a prescription drug indicated for the treatment of various cancers and hepatitis. Supported by long-term clinical evidence and substantial research, PCP is recognized as a safe and low-toxicity agent. However, as a macromolecular polysaccharide, it exhibits complex pharmacokinetic behavior following oral administration [111]. Consequently, further investigation into its higher-order structure and the associated structure–activity relationships is warranted. Table 6 summarizes the available data on other compounds’ activities and the mechanisms of their action.

## 4. Phytomedicines for the Treatment of ALD

*Tetrastigma hemsleyanum* Diels & Gilg is a herbaceous liana widely distributed in tropical and subtropical regions. It is used as the traditional Chinese medicine “San ye qing” [112]. *T. hemsleyanum* contains a variety of phenolic acids and flavonoids, including 3-caffeoylquinic acid, 5-caffeoylquinic acid, orientin, isovitexin, isoorientin, quercetin, astragalin, and kaempferol [113]. Modern pharmacological studies have shown that *T. hemsleyanum* has antioxidant, anti-inflammatory, anti-tumor, lipid-metabolism-improving, immune regulation, and hypoglycemic effects [114]. Cheng et al. found that superfine *T. hemsleyanum* powder effectively reduced the levels of ALT, AST, and ALP in the serum of ALD rats, increased the levels of SOD and GSH in the liver, and alleviated pathological changes in the liver. Superfine *T. hemsleyanum* powder also improved the imbalance of gut microbiota caused by alcohol and promoted the recovery of gut microbial diversity. Although alcohol increased *Prevotella* and decreased *Lactobacillus johnsonii* and *Raoultibacter massiliensis*, *T. hemsleyanum* effectively restored these microbiota changes [115]. Tao et al. used a Lieber–DeCarli liquid diet to establish an ALD model in C57BL/6J mice. An 80% methanol extract of *T. hemsleyanum* was used for treatment. The results showed that *T. hemsleyanum* increased the levels of antioxidant enzymes, reduced inflammatory factors in the livers of the mice, and effectively alleviated liver injury and lipid deposition. Its mechanism involved downregulating the relative levels of ACC-1 and SREBP-1c and increasing the relative levels of CPT-1A and PPARα. Metabolomics results showed that the *T. hemsleyanum* extract mainly affected fatty acid biosynthesis, β-oxidation of fatty acids, and the pentose phosphate pathway. It also significantly decreased the *Firmicutes*-to-*Bacteroidetes* ratio, reduced the abundance of harmful bacteria such as *Campilobacterota*, *Proteobacteria*, *Helicobacter*, and *Firmicutes*, and increased the abundance of beneficial bacteria such as *Bacteroidota*, *Muribaculaceae*, and *Alloprevotella* [116].

*Gentiana kurroo* Royle is an extremely endangered plant in the western and northwestern Himalayas [117] containing tannins, alkaloids, flavonoids, saponins, terpenes, and phenolics. *G. kurroo* has antioxidant, anti-inflammatory, immunomodulatory, analgesic, and antibacterial effects. Its main pharmacodynamic constituents are loganic acid, swertiamarin, gentiopicrin, gentiomarin, and gentianine [118,119,120]. Choubey et al. found that a 50% ethanol extract of *G. kurroo* reduced alcohol-induced ROS generation and the inflammatory response in Huh-7 cells. In a rat model of ALD, *G. kurroo* extract significantly increased the levels of antioxidant enzymes in the liver; alleviated liver injury and fibrosis; downregulated the expression of liver fibrosis markers (TGF-β, αSMA, and SMADs), inflammatory markers (TNF-α, IL-6, IL-1β, and NF-κB), and apoptosis markers (Caspase 3/7); and increased the levels of ADH, SREBP-1c, and mitochondrial biogenesis genes (PGC1α, Tim 23, and COX IV) in the liver [121]. The medicinal use of *G. kurroo* is primarily based on traditional experience, and toxicological and pharmacokinetic research data remain very limited. A preliminary acute toxicity study of its methanol extract indicated no observable toxicity in mice following a single oral dose of 2000 mg/kg. More in-depth investigations are warranted [122].

Pueraria lobata (*Pueraria montana* var. *lobata* (Willd.) Maesen & S. M. Almeida ex Sanjappa & Predeep) has a long history of dual use as both medicine and food in Asia. Various natural active products have been isolated and identified from Pueraria lobata, including puerarin, genistein, genistin, ononin, and soyasaponin. According to traditional Chinese medicine, Pueraria lobata can be used to treat alcoholism [123,124]. Cao et al. found that Pueraria lobata polysaccharide effectively reduced ROS, pro-inflammatory factors, lipid deposition, and fibrosis in the livers of ALD mice. Their results showed that Pueraria lobata polysaccharide increased the richness and diversity of the gut microbial community, reduced the level of *Prevotellaceae*, and increased the levels of *Bacteroidetes*, *Lactobacillus*, and *Alistipes*. In addition, Pueraria lobata polysaccharide increased the content of SCFAs in feces, thereby maintaining gut microbial homeostasis [125]. Using network pharmacology prediction, Li et al. found that puerarin and genistein are the key components of Pueraria lobata in treating ALD. In vivo studies showed that puerarin and genistein effectively alleviated liver injury, oxidative stress, and apoptosis in ALD mice. The mechanism was related to regulating the MAPK/ERK signaling pathway, increasing hepcidin expression in the liver, and reducing the production of DMT1 and FPN1 in the duodenum, thereby reducing intestinal iron absorption and improving iron overload [126]. Hu et al. found that puerarin improved lipid deposition and inflammation in the livers of ALD mice in a dose-dependent manner, and the mechanism was related to the regulation of SREBP-1c, PPARα, and NF-κB, thereby improving lipid metabolism and inflammation [127]. Puerarin also improved autophagy in the liver through the ERK/mTOR signaling pathway, alleviating liver injury in an ALD mouse model [128]. Peng et al. found that puerarin can alleviate alcohol damage to the intestinal barrier and reduce the entry of LPS into circulation, thereby inhibiting Kupffer cell activation and endotoxin receptor expression [129]. Pueraria lobata flavonoids (PLFs) are a major class of compounds of Pueraria lobata. In alcohol-injured zebrafish larvae, PLF and puerarin effectively reduced lipid deposition in the liver through a mechanism of action related to AMPKα/ACC signaling pathway regulation, leading to reduced expression of mRNA related to fatty acid synthesis (FASN and HMGCRB), ER stress (CHOP, EDEM1, GADD45αa, and ATF6), and inflammation (IL-1β, TNF-α) [130]. Zhao et al. used probiotic fermented Pueraria lobata to treat rats with ALD. The results showed that Pueraria lobata fermentation broth improved blood lipids and inflammation in the rats and increased the level of antioxidant enzymes in the liver through a mechanism related to the activation of the Nrf2/HO-1 signaling pathway. In addition, the Pueraria fermentation broth restored the gut microbiota composition of the rats and regulated the abundance of *Firmicutes*, *Bacteroidota*, *Lactobacillus*, and *Akkermansia* [131]. A clinical study showed that compared to placebo, administering adult male heavy drinkers 250 mg/d of Pueraria lobata extract orally significantly reduced weekly alcohol consumption and the number of days of heavy drinking, with good medication adherence [132].

*Penthorum chinense* Pursh is a traditional Chinese medicine used to treat liver diseases. Its main components include kaempferol, quercetin, 5-hydroxy-flavanone-7-O-D-glucoside, pinocembrin, and catechins [133]. Several studies have shown that *P. chinense* can significantly improve ALD. Zhang et al. found that *P. chinense* improved blood lipids and liver damage in ALD mice and regulated glycine, serine and threonine metabolism; glutathione metabolism; arginine and proline metabolism; alanine, aspartate and glutamate metabolism; butanoate metabolism; and primary bile acid biosynthesis. In addition, *P. chinense* adjusted the *Firmicutes*-to-*Bacteroidetes* ratio and improved the intestinal flora balance in mice with ALD [134]. Zhao et al. showed that *P. chinense* effectively reduced ROS and lipid deposition due to alcohol injury in the livers of zebrafish larvae. Its mechanism of action was related to activating the AMPK/mTOR and Nrf2 signaling pathways, thereby improving liver antioxidant levels, autophagy, and lipid metabolism [135]. Using a combination of network pharmacology and experimental verification, Jiang et al. found that *P. chinense* may exert a therapeutic effect in ALD through the PI3K/Akt signaling pathway, thereby alleviating oxidative stress, lipid deposition, and apoptosis in the liver. Quercitrin is an important pharmacodynamic component of *P. chinense* [136]. Li et al. also used network pharmacology combined with experimental verification to study *P. chinense*, finding that it down-regulated the Ras/Raf/MEK/ERK pathway, thereby improving the antioxidant capacity of the liver and alleviating inflammation. Their prediction indicated that quercetin, apigenin, and thonningianin B may be the effective components of *P. chinense* [137]. *P. chinense* is a plant representative of the concept of “homology between medicine and food”. In-depth research has confirmed its hepatoprotective activity and overall favorable safety profile [138]. Consequently, it may be a promising botanical drug candidate for treating ALD.

*Prunella vulgaris* L. is a perennial herb. It is medicinal and edible, and its dry fruit spikes are consumed for health purposes. *P. vulgaris* contains complex and diverse chemical components—mainly triterpenoids, sterols, flavonoids, phenylpropanoids, volatile oils, organic acids, and polysaccharides [139]. In traditional and modern applications, *P. vulgaris* exhibits hepatoprotective effects and can reduce liver injury and fibrosis caused by various factors [140,141]. Rao et al. found that *P. vulgaris* regulated PPARα and CPT-1 in the liver in an ALD mouse model, promoting fatty acid oxidation and thus alleviating lipid deposition. In addition, *P. vulgaris* alleviated the intestinal barrier damage caused by alcohol and reduced endotoxin translocation, inhibiting TLR4/Myd88 signaling pathway-mediated liver inflammation. *P. vulgaris* also improved gut microbiota dysbiosis and reversed alcohol-induced changes in Parabacteroides, Shigella, Coriobacteriaceae, Clostridiales, and Lachnospiraceae at the genus level [142]. Prunella vulgaris, a traditional herb used for both medicinal and culinary purposes, is considered non-toxic. This was supported by an acute toxicity study showing that its extract has an acute oral LD_50_ of more than 21.5 g/kg body weight in both female and male mice, categorizing it as “non-toxic” according to standard classification [143]. Table 7 summarizes the available data on phytomedicines’ activities and the mechanisms of their action.

## 5. Other Natural Products

Sea cucumber is a nutritionally rich and important food and medicine resource. Various sea cucumber extracts have demonstrated antioxidant and lipid metabolism-regulating effects [144]. Song et al. found that sea cucumber sulfated polysaccharide extracted from *Stichopus japonicu* can effectively reduce the levels of TC and TG in serum and MDA in the liver, thus alleviating acute alcoholic liver injury in mice. A metabolomics study revealed that the ameliorative effect of sea cucumber sulfated polysaccharide on alcoholic liver injury was related to glycerophospholipid metabolism regulation [145]. Zhu et al. found that sea cucumber peptide extracted from *Apostichopus japonicus* significantly improved alcohol-induced inflammation and lipid deposition in mouse livers. Sea cucumber peptide can activate the Nrf2/HO-1 pathway and reduce the nuclear translocation of NF-κB. In addition, sea cucumber peptide regulates the levels of PGC-1α, OPA1, MFN2, and Drp1, thereby improving mitochondrial dynamics [146]. Wang et al. found that sea cucumber etherphospholipids can effectively reduce oxidative stress, mitochondrial damage, and lipid deposition in the liver of mice caused by alcohol. Sea cucumber etherphospholipids reduced the expression of CD36, FATP1, and DGAT1 in a liver injured by alcohol, thereby reducing fatty acid uptake and TG synthesis in the liver [147]. As a traditional food material, it is generally considered safe at the conventional dosage, but systematic research on the pharmacokinetic parameters of its complex components is still lacking. In the future, rigorous clinical trials should be designed to verify the efficacy and safety of its promising active ingredients.

*Protaetia brevitarsis* is an insect belonging to the Coleoptera Scarabaeidae family. Its larvae have been used as medicine in East Asia. *P. brevitarsis* has been reported to have multiple effects, such as antioxidant, anticancer, hypoglycemic, and hepatoprotective [148]. Lee et al. found that *P. brevitarsis* could significantly enhance the activity of ADH and ALDH in HepG2 cells and improve the viability of alcohol-injured HepG2 cells. In an acute alcohol-induced mouse model, *P. brevitarsis* significantly improved alcohol metabolism and decreased ethanol and acetaldehyde concentrations in the blood. In the chronic alcohol-induced mouse model, *P. brevitarsis* effectively improved blood lipids, liver antioxidant capacity, and mitochondrial function. First, *P. brevitarsis* regulates p-AMPK, SREBP-1a, SREBP-2, PPARγ, C/EBPα, and FAS, improving liver lipid metabolism. Second, *P. brevitarsis* regulates TLR4, MyD88, p-IκBα, p-NF-κB, IL-1β, and TNF-α, reducing liver inflammation. Finally, *P. brevitarsis* regulates TGF-β1, p-Smad2, p-Smad3, MMP-1, and MMP-2, alleviating liver fibrosis. In conclusion, *P. brevitarsis* alleviates liver injury by regulating multiple pathways [149].

Bear bile powder is a traditional Chinese medicine used to treat liver dysfunction. Due to ethical and animal protection issues, the procurement of natural bear bile has become very complicated and controversial. As a result, chicken bile is often biotransformed to produce cultured bear bile powder, a synthetic alternative to natural bear bile [150]. Studies have shown that synthetic bear bile powder can reduce liver inflammation and improve lipid metabolism, with a good therapeutic effect in a variety of liver diseases [151,152]. Wang et al. showed that bear bile powder reduced the duration of alcohol-induced inebriation and lipid levels in serum, increased the levels of liver antioxidant enzymes, and alleviated pathological damage to the liver. An investigation of gut microbiota showed that bear bile powder improved the diversity and composition of gut microbiota. Statistical analysis of the differences between groups showed that alcohol significantly reduced the abundance of *Lachnospiraceae* NK4A136 in gut microbiota and significantly increased the abundance of *Escherichia-Shigella*. Notably, bear bile powder administration significantly restored gut microbial homeostasis. Metabolomic analysis showed that the therapeutic effect of bear bile powder may be related to the regulation of glycerophospholipid metabolism, alpha-linolenic acid metabolism, retinol metabolism, and caffeine metabolism. In addition, a network medicine framework analysis found that chenodeoxycholic acid and ursodeoxycholic acid may be the most important components in improving ALD [153]. Table 8 summarizes the available data on other natural products’ activities and their mechanisms of action.

## 6. Discussion

ALD has emerged as one of the most common liver diseases, and its prevalence continues to increase. Complete alcohol abstinence is the cornerstone of ALD treatment; however, given the addictive nature of alcohol, achieving this goal is often a formidable challenge. Consequently, a comprehensive therapeutic approach is warranted in the treatment of ALD that encompasses gradual alcohol abstinence while employing hepatoprotective medications to restore liver health. Numerous natural products have been extensively researched, and in this article, we reviewed preclinical studies reporting natural products with beneficial effects on ALD. However, further research is needed to ascertain their clinical applicability in ALD treatment.

The occurrence and progression of ALD involve multiple mechanisms, with the main cause being alcohol-induced oxidative stress and inflammation, ultimately leading to liver fibrosis. This article explores how natural products exert therapeutic effects in ALD by regulating oxidative stress, inflammation, lipid metabolism, autophagy, and gut microbiota. Notably, some natural products, especially phytomedicines, comprise many compounds and can exert effects through multiple pathways and targets in ALD treatment. It is imperative to conduct in-depth, systematic studies using transcriptomics, proteomics, and pathway inhibitors to determine their mechanisms. In addition, while existing research has mainly focused on their anti-inflammatory properties, other aspects, such as their ability to improve lipid deposition, anti-fibrotic effects, and intricate interplay with gut microbiota, remain relatively underexplored. In this regard, gut microbiota transplantation studies can serve as a valuable tool in revealing the influence of gut bacteria in ALD, potentially providing novel insights into the multifaceted therapeutic mechanisms of natural products.

This review examines the potential benefits of these natural compounds in preventing and treating the progression of ALD. Several of these compounds, including quercetin, berberine, glycyrrhizic acid, and Poria cocos polysaccharide, show promising therapeutic potential. They have been investigated to varying degrees in clinical studies, with substantial research supporting their positive therapeutic effects against ALD. The mechanisms of action and current levels of evidence for these compounds are summarized in Table 9. This review found other natural products that also demonstrate beneficial effects against ALD. Their common mechanisms include anti-inflammatory and antioxidant activities, as well as reducing lipid accumulation. The research on monomeric compounds is more definitive, with clear toxicological and pharmacokinetic parameters established. In contrast, botanical or animal-derived preparations, due to their complex composition, require further toxicological and pharmacokinetic investigations to substantiate their efficacy.

While natural products show promising therapeutic potential for ALD, their concomitant use with conventional medications raises significant concerns regarding herb-drug interactions. For instance, berberine is a known inhibitor of both CYP3A4 and P-glycoprotein. This activity may alter the plasma concentrations of commonly prescribed drugs metabolized by the same pathways, such as statins, potentially increasing toxicity or altering therapeutic efficacy [154]. Similarly, the primary active constituents of *Penthorum chinense* Pursh are flavonoids, a class of compounds frequently associated with the inhibition of hepatic drug-metabolizing enzymes, particularly cytochrome P450 isoforms like CYP3A4 and CYP2C9. Concomitant use could theoretically slow the clearance of co-administered drugs relying on these enzymes, leading to abnormally elevated plasma levels and an increased risk of adverse effects. Examples include statins and calcineurin inhibitor tacrolimus [155,156]. Therefore, research on these promising natural products should be expanded to include systematic drug interaction studies, accompanied by therapeutic drug monitoring and assessments of hepatic and renal function in clinical settings.

In summary, natural products offer a rich source of potential therapeutic agents for ALD, with diverse mechanisms of action that target multiple aspects of the disease pathophysiology. Although promising, their clinical translation requires rigorous scientific validation through well-designed studies that address bioavailability, standardization, and safety concerns. The integration of traditional knowledge with modern scientific approaches holds great promise for developing effective, safe, and accessible treatments for alcoholic liver disease.

## 7. Methodology

In this review, we searched the PubMed database (https://pubmed.ncbi.nlm.nih.gov/) and Google Scholar (https://scholar.google.com/), accessed on 28 December 2025. The search keywords included “(alcoholic liver disease) OR (alcoholic liver injury)”, “((alcoholic liver disease) OR (alcoholic liver injury)) AND (NP)”, “(Clinical) AND (NP)”, “(Toxicology) AND (NP)”, “(Pharmacokinetics) AND (NP)”, where “NP” was replaced with the name of each natural product investigated.

## Figures and Tables

**Table 1:** Mechanisms of flavonoids in the treatment of ALD.

Natural Product	Model	Route/Duration of Administration In Vivo	Function	Mechanism/Target	Reference
Quercetin	In vivo: Mice were fed a Lieber–DeCarli liquid diet In vitro: Alcohol injury to L02 cells	Oral 100 mg/kg body weight; 12 weeks	Anti-aging Lipid-lowering	purine metabolism pathway p53, p21, p16	[39]
	In vivo: Mice were fed a Lieber–DeCarli liquid diet	Oral 100 mg/kg body weight; 12 weeks	Lipid-lowering Improves lipophagy	AMPK, LC3II, PLIN2	[40]
	In vivo: Mice were fed a Lieber–DeCarli liquid diet	Oral 100 mg/kg body weight; 12 weeks	Improves mitophagy	AMPK, ERK2, Parkin, VDAC1, FoxO3a	[41]
	In vitro: Alcohol injury to L02 cells	-	Antioxidant Lipid-lowering Anti-pyroptosis	OPA1, MFN1, MFF, Drp1, Fis1, PGC-1α, NLRP3 inflammasome	[42]
	In vivo: Rats were gavaged with alcohol	Oral 100 mg/kg body weight; 14 days	Antioxidant Anti-inflammatory	Nrf2/HO-1 signaling pathway NLRP3 inflammasome	[43]
	In vivo: Mice were fed a Lieber–DeCarli liquid diet In vitro: Alcohol injury to HepG2 cells	Oral 100 mg/kg body weight; 12 weeks	Improves mitophagy Reduces iron accumulation Protects mitochondria	PINK1, Parkin, BNIP3, LC3II, p62, TOM20, VDAC1, frataxin	[44]
	In vivo: Mice were fed a Lieber–DeCarli liquid diet In vitro: Alcohol injury to AML12 cells	Oral 100 mg/kg body weight; 12 weeks	Anti-ER stress Anti-ferroptosis	PERK signaling pathway Gpx4, xCT, ACSL4	[45]
	In vivo: Mice were fed a Lieber–DeCarli liquid diet	Oral 100 mg/kg body weight; 12 weeks	Anti-ferroptosis Improves ferritinophagy	p62, LC3II, NCOA4, FOXO1	[46]
	In vivo: Alcohol-soaked zebrafish larvae	Soak 25 μM, 50 μM, 100 μM; 48 h	Antioxidant Lipid-lowering	PI3K/Keap1/Nrf2 signaling pathway P2X7r	[47]
Cyanidin-3-O-β-glucoside	In vivo: Mice were fed a Lieber–DeCarli liquid diet	Oral 100 mg/kg body weight; 7 weeks	Antioxidant Lipid-lowering Protects the intestinal barrier Gut microbiota regulation	*Firmicutes*, *Bacteroidetes*, *Muribaculaceae*, *Bacteroides*, *Collinsella*, *Escherichia-Shigella*, *Blautia*, *Ruminococcus*, *Enterococcus*, *Prevotella*, *Romboutsia*, *Streptococcus*, *Bilophila*, *Methylobacterium-Methylorubrum*	[52]
	In vivo: Mice were fed a Lieber–DeCarli liquid diet In vitro: Alcohol injury to AML12 cells	Intraperitoneal 50 μg/kg body weight; 14 days	Antioxidant Anti-inflammatory Lipid-lowering Improves mitophagy	SAA1, 4-HNE, AMPK, PINK1, Parkin, LC3II, TOM20	[53]
	In vivo: Mice were provided drinking water supplemented with alcohol	Oral 200 mg/kg body weight; 14 days	Anti-inflammatory Lipid-lowering	SIRT1, NF-κB NLRP3 inflammasome	[54]

**Table 2:** Mechanisms of alkaloids in the treatment of ALD.

Natural Product	Model	Route/Duration of Administration In Vivo	Function	Mechanism/Target	Reference
Capsaicin	In vivo: Mice were gavaged with alcohol	Oral 10 and 20 mg/kg body weight; 10 days	Antioxidant Anti-inflammatory Protect mitochondria	NF-κB, MMP2, MMP9, TIMP1	[60]
	In vivo: Mice were gavaged with alcohol In vitro: Alcohol injury to AML-12 cells	Oral 10 mg/kg body weight; 14 days	Anti-pyroptosis	NLRP3 inflammasome TRPV1, CHMP4B	[61]
Berberine	In vivo: Mice were fed a Lieber–DeCarli liquid diet	Oral 120 mg/kg body weight; 5 weeks	Antioxidant Lipid-lowering	CYP2E1, PPARα, PGC-1α, HNF4α, MTTP	[65]
	In vivo: Rats were gavaged with alcohol	Oral 200 mg/kg body weight; 5 weeks	Anti-inflammatory Lipid-lowering	THRSP, ACC, FASN, ACLY, PPARα, CPT1α, ACOX1	[66]
	In vivo: Mice were fed a Lieber–DeCarli liquid diet In vitro: Alcohol injury to AML-12 cells	Oral 50, 100 and 200 mg/kg body weight; 14 days	Lipid-lowering	AMPK/SIRT1 signaling pathway SREBP-1c, SREBP-2, FASN, HMGCR	[67]
	In vivo: Mice were gavaged with alcohol	Oral 10, 50 and 100 mg/kg body weight; 33 days	Gut microbiota regulation	*Verrucomicrobia*, *Terrisporobacter*, *Helicobacter*, *Pseudoflavonifractor*, *Mucisirillum*, *Ruminiclostridium*, *Alistipes*, *Lachnoclostridium*	[68]

**Table 3:** Mechanisms of terpenoids in the treatment of ALD.

Natural Product	Model	Route/Duration of Administration In Vivo	Function	Mechanism/Target	Reference
Glycyrrhizic acid	In vivo: Mice were fed a liquid alcohol diet In vitro: FFA and alcohol damage the THP-1 and HepG2 co-culture system	Oral 20 mg/kg body weight; 10 days	Antioxidant Anti-inflammatory Anti-pyroptosis Lipid-lowering	SHP1/SYK signaling pathway NOX2, NOX3, Nrf2, STING, p-PDE4B, NLRP3, IL-1β, GSDMD, Caspase-1, Caspase-4	[74]
	In vivo: Mice were fed a Lieber–DeCarli liquid diet In vitro: Alcohol injury to AML-12 cells	Oral 12.5 and 25 mg/kg body weight; 10 days	Anti-inflammatory Lipid-lowering	DDX5/STAT1 signaling pathway FASN, SCD1, SREBP-1c	[75]
Asperosaponin VI	In vivo: Mice were fed a Lieber–DeCarli liquid diet	Oral 10 and 20 mg/kg body weight; 4 weeks	Antioxidant Anti-inflammatory Anti-fibrotic Anti-ER stress Lipid-lowering	AMPK signaling pathway PERK/ELF2 signaling pathway	[79]
Hederagenin	In vivo: Rats were gavaged with alcohol	Oral 50 mg/kg body weight; 21 days	Lipid-lowering Anti-inflammatory Anti-apoptosis	TNF-α, IL-6, COX-2, Bax, Bcl-2, p53, Akt signaling pathways ERK signaling pathways	[82]

**Table 4:** Mechanisms of phenylpropanoids in the treatment of ALD.

Natural Product	Model	Route/Duration of Administration In Vivo	Function	Mechanism/Target	Reference
Nodakenin	In vivo: Mice were fed a Lieber–DeCarli liquid diet In vitro: Alcohol injury to AML-12 or HepG2 cells	Oral 10 and 20 mg/kg body weight; 4 weeks	Anti-inflammatory Lipid-lowering Anti-pyroptosis	NLRP3 inflammasome Nur77/P2X7r signaling pathway PPARα, SREBP-1, Lipin-1	[85]
Glycycoumarin	In vivo: Mice were fed a Lieber–DeCarli liquid diet In vitro: Alcohol injury to AML-12 or HepG2 cells	Intraperitoneal 10 and 20 mg/kg body weight; 3 weeks	Antioxidant Improves autophagy	p38 MAPK/Nrf2/HO-1 signaling pathway LC3II, p62	[87]
α-Asarone	In vivo: Mice were fed a Lieber–DeCarli liquid diet	Intraperitoneal 5 and 10 mg/kg body weight; 10 days	Antioxidant Anti-inflammatory Anti-apoptosis Improves autophagy	AMPK signaling pathway Beclin-1, LC3, p53	[90]

**Table 5:** Mechanisms of steroids in the treatment of ALD.

Natural Product	Model	Route/Duration of Administra-tion In Vivo	Function	Mechanism/Target	Reference
Dioscin	In vivo: Mice were fed a Lieber–DeCarli liquid diet	Oral 20, 40 and 80 mg/kg body weight; 10 days	Anti-inflammatory Anti-fibrotic	TLR4/MyD88 signaling pathway TGF-β/Smad signaling pathway NF-κB, αSMA, Collagen I	[93]
	In vivo: Mice and Rats were fed an ethanol-containing liquid diet	Oral 28, 56 and 84 mg/kg body weight; 2 weeks	Antioxidant Anti-ER stress Anti-inflammatory Anti-apoptosis Lipid-lowering	MAPK signaling pathway GRP78, ATF6, eIF2, NF-κB, p53, Caspase-3, Caspase-9, PARP, PPARα, MCAD, ACSL1, ACSL5, ACADS, ALDH7A1	[94]
Daucosterol	In vivo: Mice were fed a Lieber–DeCarli liquid diet In vitro: Alcohol injury to HepG2 cells	Oral 10 mg/kg body weight; 10 days	Antioxidant Anti-inflammatory Lipid-lowering Anti-fibrotic	Nrf2 signaling pathway p38 MAPK/NF-κB signaling pathway NLRP3 inflammasome FASN, SREBP-1c, SCD1, Collagen1, Collagen 3A1, αSMA	[98]

**Table 6:** Mechanisms of other compounds in the treatment of ALD.

Natural Product	Model	Route/Duration of Administration In Vivo	Function	Mechanism/Target	Reference
Ligustilide	In vivo: Mice were fed a Lieber–DeCarli liquid diet In vitro: Alcohol injury to AML-12 cells LPS/ATP stimulates MPMs	Oral 15, 30 and 60 mg/kg body weight; 10 days	Anti-inflammatory Lipid-lowering Anti-pyroptosis	PPARα, SREBP-1, RXFP1 NLRP3 inflammasome	[103]
*Poria cocos* polysaccharide	In vivo: Mice were fed a Lieber–DeCarli liquid diet	Oral 50 and 100 mg/kg body weight; 4 weeks	Anti-inflammatory Lipid-lowering Protects the intestinal barrier Gut microbiota regulation	TLR4/ NF-κB signaling pathway FXR/FGF15/CYP7A1 signaling pathway *Parabacteroides distasonis*	[108]
	In vivo: Rats were gavaged with alcohol In vitro: Alcohol injury to BRL-3A cells	Oral 100 mg/kg body weight; 6 weeks	Antioxidant Anti-inflammatory Anti-ferroptosis	Nrf2 signaling pathway NF-κB signaling pathway FTH1, Gpx4	[109]
	In vivo: Mice were fed a Lieber–DeCarli liquid diet	Oral 25, 50 and 100 mg/kg body weight; 10 days	Antioxidant Anti-inflammatory Anti-apoptosis Lipid-lowering Protects the intestinal barrier	TLR4/NF-κB signaling pathway MAPK signaling pathway	[110]

**Table 7:** Mechanisms of phytomedicines in the treatment of ALD.

Natural Product	Model	Route/Duration of Administration In Vivo	Function	Mechanism/Target	Reference
*Tetrastigma hemsleyanum* Diels & Gilg	In vivo: Rats were gavaged with alcohol	Oral extract 0.25, 0.5 and 1.0 g/kg body weight; 6 weeks	Antioxidant Gut microbiota regulation	*Prevotella*, *Lactobacillus johnsonii*, *Raoultibacter massiliensis*	[115]
	In vivo: Mice were fed a Lieber–DeCarli liquid diet	Oral extract 100 and 500 mg/kg body weight; 10 days	Antioxidant Anti-inflammatory Lipid-lowering Gut microbiota regulation	ACC-1, SREBP-1c, CPT-1A, PPARα *Campilobacterota*, *Firmicutes*, *Proteobacteria*, *Helicobacter*, *Bacteroidota*, *Muribaculaceae*, *Alloprevotella*	[116]
*Gentiana kurroo* Royle	In vivo: Rats were gavaged with alcohol and a single intraperitoneal low dose of CCl_4_ In vitro: Alcohol injury to Huh-7 cells	Oral extract 100, 200 and 400 mg/kg body weight; 15 days	Antioxidant Anti-inflammatory Anti-apoptosis Anti-fibrotic	TGF-β, αSMA, SMADs, TNF-α, IL-6, IL-1β, NF-κB, Caspase 3/7, ADH, SREBP-1c, PGC1α, Tim 23, COX IV	[121]
Pueraria lobata	In vivo: Mice were gavaged with alcohol	Oral polysaccharide 100, 200 and 400 mg/kg body weight; 21 days	Antioxidant Anti-inflammatory Anti-fibrotic Lipid-lowering Gut microbiota regulation	*Prevotellaceae*, *Bacteroidetes*, *Lactobacillus*, *Alistipes*	[125]
	In vivo: Mice were gavaged with alcohol	Oral puerarin 0.3 mmoL/kg body weight; 8 weeks	Antioxidant Anti-apoptosis Reduces iron overload	MAPK/ERK signaling pathway hepcidin, DMT1, FPN1	[126]
	In vivo: Mice were fed a Lieber–DeCarli liquid diet In vitro: Alcohol injury to AML12 cells	Oral puerarin 25, 50 and 100 mg/kg body weight; 10 days	Anti-inflammatory Lipid-lowering	SREBP-1c, PPARα, NF-κB	[127]
	In vivo: Mice were fed a Lieber–DeCarli liquid diet	Intravenously puerarin 42 mg/kg body weight; 2 weeks	Improves autophagy	ERK/mTOR signaling pathway	[128]
	In vivo: Rats were fed a Lieber–DeCarli liquid diet	Oral puerarin 90 and 180 mg/kg body weight; 5 weeks	Anti-inflammatory Protects the intestinal barrier	ZO-1, CD68, LBP, CD14, TLR2, TLR4	[129]
	In vivo: Alcohol-soaked zebrafish larvae	Soak in extract 0.05, 0.1 and 0.2 mg/mL 48 h	Lipid-lowering Anti-inflammatory Anti-ER stress	AMPKα/ACC signaling pathway FASN, HMGCRB, CHOP, EDEM1, GADD45αa, ATF6, IL-1β, TNF-α	[130]
	In vivo: Rats were gavaged with alcohol	Oral fermentation broth 10 mL/kg body weight; 5 weeks	Antioxidant Anti-inflammatory Lipid-lowering Gut microbiota regulation	Nrf2/HO-1 signaling pathway *Firmicutes*, *Bacteroidota*, *Lactobacillus*, *Akkermansia*	[131]
*Penthorum chinense* Pursh	In vivo: Mice were gavaged with alcohol	Oral extract 0.29, 0.87 and 2.61 g/kg body weight; 12 days	Lipid-lowering Gut microbiota regulation	Glycine, serine and threonine metabolism Glutathione metabolism Arginine and proline metabolism Alanine, aspartate and glutamate metabolism Butanoate metabolism Primary bile acid biosynthesis	[134]
	In vivo: Alcohol-soaked zebrafish larvae	Soak in extract 25, 50 and 100 μg/mL 48 h	Antioxidant Improves autophagy Lipid-lowering	AMPK/mTOR signaling pathways Nrf2 signaling pathways	[135]
	In vivo: Mice were gavaged with alcohol	Oral extract 2, 4 and 8 g crude drug/kg body weight; 12 days	Antioxidant Lipid-lowering Anti-apoptosis	PI3K/Akt signaling pathway	[136]
	In vivo: Mice were fed a Lieber–DeCarli liquid diet	Oral extract 1.25 and 2.5 g crude drug/kg body weight; 21 days	Antioxidant Anti-inflammatory	Ras/Raf/MEK/ERK pathway	[137]
*Prunella vulgaris* L.	In vivo: Mice were fed a Lieber–DeCarli liquid diet	Oral extract 150 and 300 mg/kg body weight; 4 weeks	Anti-inflammatory Lipid-lowering Protects intestinal barrier Gut microbiota regulation	PPARα and CPT-1 TLR4/Myd88 signaling pathway	[142]

**Table 8:** Mechanisms of other natural products in the treatment of ALD.

Natural Product	Model	Route/Duration of Administration In Vivo	Function	Mechanism/Target	Reference
Sea cucumber sulfated polysaccharide	In vivo: Mice were gavaged with alcohol	Oral 60 and 360 mg/kg body weight; 5 days	Antioxidant Lipid-lowering	Glycerophospholipid metabolism	[145]
Sea cucumber peptide	In vivo: Mice were gavaged with alcohol In vitro: Alcohol injury to L02 cells	Oral 10 and 20 mg/kg body weight; 35 days	Antioxidant Anti-inflammatory Lipid-lowering Protects mitochondria	Nrf2/HO-1 signaling pathway NF-κB, PGC-1α, OPA1, MFN2, Drp1	[146]
Sea cucumber etherphospholipids	In vivo: Mice were fed a AIN93 liquid diet	Oral feed containing 0.3% ether-phospholipid 5 weeks	Antioxidant Lipid-lowering Protects mitochondria	CD36, FATP1, DGAT1	[147]
*Protaetia brevitarsis*	In vivo: Mice were gavaged with alcohol In vitro: Alcohol injury to HepG2 cells	Oral extract 50 and 100 mg/kg body weight; 8 weeks	Antioxidant Lipid-lowering Protects mitochondria Anti-inflammatory Anti-fibrotic	p-AMPK, SREBP-1a, SREBP-2, PPARγ, C/EBPα, FAS, TLR4, MyD88, p-IκBα, p-NF-κB, TNF-α, IL-1β, TGF-β1, p-Smad2, p-Smad3, MMP-1, MMP-2	[149]
Synthetic bear bile powder	In vivo: Mice were gavaged with alcohol	Oral 100 and 500 mg/kg body weight; 15 days	Antioxidant Lipid-lowering Gut microbiota regulation	*Lachnospiraceae* NK4A136, *Escherichia-Shigella* glycerophospholipid metabolism alpha-linolenic acid metabolism retinol metabolism caffeine metabolism	[153]

**Table 9:** Comparison of the most promising compounds for treating ALD.

Natural Product	Class	Current Evidence Level	Mechanism of Treating ALD	Recognized Problems
Quercetin	Flavonoid	Used for dietary supplements; application of preclinical studies in ALD	Anti-aging; lipid-lowering; improve lipophagy; improve mitophagy; antioxidant; anti-pyroptosis; anti-inflammatory; protects mitochondria; anti-ER stress; anti-ferroptosis; improves ferritinophagy	Oral bioavailability is extremely low
Berberine	Alkaloid	Clinical application in the treatment of intestinal infections; application of preclinical studies in ALD	Antioxidant; lipid-lowering; anti-inflammatory; gut microbiota regulation	Oral bioavailability is extremely low Causes gastrointestinal discomfort
Glycyrrhizic acid	Terpenoid	Clinical application in the treatment of liver disease	Antioxidant; anti-inflammatory; anti-pyroptosis; lipid-lowering	Long-term use leads to abnormal elevation of cortisol levels
*Poria cocos* polysaccharide	polysaccharide	Clinical application in adjuvant therapy for tumors; application of preclinical studies in ALD	Anti-inflammatory; lipid-lowering; antioxidant; anti-ferroptosis; anti-apoptosis; protects intestinal barrier; gut microbiota regulation	Unclear pharmacokinetics

## Data Availability

No new data were created or analyzed in this study. Data sharing is not applicable to this article.
